# EZH2 and Endometrial Cancer Development: Insights from a Mouse Model

**DOI:** 10.3390/cells11050909

**Published:** 2022-03-07

**Authors:** Xin Fang, Nan Ni, Xiaofang Wang, Yanan Tian, Ivan Ivanov, Monique Rijnkels, Kayla J. Bayless, John P. Lydon, Qinglei Li

**Affiliations:** 1 Department of Veterinary Integrative Biosciences, Texas A&M University, College Station, TX 77843, USA; xfang@cvm.tamu.edu (X.F.); nni@cvm.tamu.edu (N.N.); mrijnkels@cvm.tamu.edu (M.R.); 2 Department of Biomedical Sciences, Texas A&M University College of Dentistry, Dallas, TX 75246, USA; xfwang@tamu.edu; 3 Department of Veterinary Physiology and Pharmacology, Texas A&M University, College Station, TX 77843, USA; ytian@cvm.tamu.edu (Y.T.); iivanov@cvm.tamu.edu (I.I.); 4 Department of Molecular and Cellular Medicine, Texas A&M University Health Science Center, Bryan, TX 77807, USA; kaylajb@tamu.edu; 5 Department of Molecular and Cellular Biology, Baylor College of Medicine, Houston, TX 77030, USA; jlydon@bcm.edu

**Keywords:** *Ezh2*, endometrial cancer, *Pten*, mouse model

## Abstract

Enhancer of zeste homolog 2 (EZH2), a core component of polycomb repressive complex 2, plays an important role in cancer development. As both oncogenic and tumor suppressive functions of EZH2 have been documented in the literature, the objective of this study is to determine the impact of *Ezh2* deletion on the development and progression of endometrial cancer induced by inactivation of phosphatase and tensin homolog (*PTEN*), a tumor suppressor gene frequently dysregulated in endometrial cancer patients. To this end, we created mice harboring uterine deletion of both *Ezh2* and *Pten* using Cre recombinase driven by the progesterone receptor (*Pgr*) promoter. Our results showed reduced tumor burden in *Pten^d/d^; Ezh2^d/d^* mice compared with that of *Pten^d/d^* mice during early carcinogenesis. The decreased Ki67 index in EZH2 and PTEN-depleted uteri versus that in PTEN-depleted uteri indicated an oncogenic role of EZH2 during early tumor development. However, mice harboring uterine deletion of both *Ezh2* and *Pten* developed unfavorable disease outcome, accompanied by exacerbated epithelial stratification and heightened inflammatory response. The observed effect was non-cell autonomous and mediated by altered immune response evidenced by massive accumulation of intraluminal neutrophils, a hallmark of endometrial carcinoma in *Pten^d/d^; Ezh2^d/d^* mice during disease progression. Hence, these results reveal dual roles of EZH2 in endometrial cancer development.

## 1. Introduction

Endometrial cancer is the most common cancer in the genital tract in women, with approximately 65,570 new cases and 12,940 deaths each year in the United States [1]. Endometrial cancer is classified into two distinct types [2]. The type I cancer represents the major type (~90%) and is often companied by endometrial hyperplasia [2,3]. The type II cancer accounts for ~10% of the total cases and is more aggressive than the type I cancer [2,3,4,5]. Histologically, the type I cancer is endometrioid carcinoma while the type II cancer consists of several subtypes, including serous carcinoma and clear-cell carcinoma [6]. Notably, the type I, but not the type II, endometrial cancer is related to estrogen stimulation [7]. Using molecular sequencing technologies, endometrial cancer has been classified into the following types by The Cancer Genome Atlas (TCGA) Research Network: DNA polymerase epsilon catalytic subunit (*POLE*) (ultramutated), microsatellite-instability (MSI) (hypermutated), copy-number low, as well as copy-number high [8]. To facilitate the classification in clinical practice, the Proactive Molecular Risk Classifier for Endometrial Cancer (ProMisE) has been developed and validated, with the inclusion of immunohistochemical analysis of DNA mismatch repair (MMR) protein and tumor protein p53 (TP53) [9,10,11]. Interestingly, a recent report shows that a combination of tumor-infiltrating lymphocytes pattern and MMR may be used as a surrogate for the *POLE* mutation group [12]. ProMisE has been used in molecular diagnosis of human endometrial cancer [13].

Significant challenges remain for endometrial cancer treatment. Determining the histological subtype of endometrial cancer is an effective strategy that guides cancer treatment, with an emerging need to incorporate more molecular details into clinical interventions [14]. While surgery remains to be the most common option to treat this gynecological malignancy, new therapeutic strategies targeting actionable mutations and/or molecular pathways are potentially valuable [15,16]. Of particular importance, knowledge gaps need to be filled in areas of early cancer diagnostics, cancer risk stratification, and molecular identity-based treatment options [14].

Phosphatase and tensin homolog (*PTEN*), a tumor suppressor gene, is frequently dysregulated in the type I endometrial cancer patients [2]. Loss of heterozygosity of chromosome 10q where *PTEN* is located (chromosome 10q23.3) or intragenic mutation of *PTEN* has been identified in endometrial cancer [2,17,18,19]. Conditional deletion of *Pten* in the mouse uterus promotes endometrial cancer development, lending credence to the role of PTEN in the pathogenesis of endometrial cancer [20]. Dysregulation of the phosphatidylinositol 3-kinase (PI3K) pathway, mitogen-activated protein kinase (MAPK) pathway, catenin beta 1 (CTNNB1), or AT-rich interaction domain 1A (ARID1A or BAF250) appears common in endometrial cancer patients [21]. Meanwhile, mutations in phosphatidylinositol-4,5-bisphosphate 3-kinase catalytic subunit alpha (*PIK3CA*), phosphoinositide-3-kinase regulatory subunit 1 (*PIK3R1*), KRAS proto-oncogene, GTPase (*KRAS*), fibroblast growth factor receptor 2 (*FGFR2*), protein phosphatase 2 scaffold subunit Aalpha (*PPP2R1A*), and tumor protein p53 (*TP53*) have also been identified in endometrioid carcinoma and serous endometrial cancer [21].

Enhancer of zeste homolog 2 (EZH2) is a core component of polycomb repressive complex 2 (PRC2) [22]. EZH2 is a well-established histone methyltransferase that regulates gene expression via inducing the tri-methylation of lysine 27 on histone H3 (H3K27) [23]. EZH2 is overexpressed in both human endometrial cancer cell lines and endometrial cancer tissues [24]. Moreover, gain-of-function [25,26] or loss-of-function [27,28] mutations of EZH2 frequently occur in cancers [23,29]. Of note, both tumor-promoting and tumor-suppressive effects of EZH2 have been documented in cancer development [29]. However, the role of EZH2 in endometrial cancer remains poorly defined.

We previously showed that uterine-specific loss of EZH2 in the mouse provokes the formation of stratified epithelia and the development of endometrial hyperplasia [30]. To determine the impact of *Ezh2* deletion on the development and progression of endometrial cancer induced by PTEN inactivation, we generated mice containing double deletion of *Ezh2* and *Pten* using Cre recombinase driven by the progesterone receptor (*Pgr*) promoter. Our results revealed dual roles of EZH2 in endometrial cancer development.

## 2. Materials and Methods

### 2.1. Animals and Ethics

Protocols involving the use of mice were approved by Texas A&M University Institutional Animal Care and Use Committee. Mice were on a mixed C57BL/6/129SvEv background and handled according to NIH guideline for the Care and Use of Laboratory Animals. The reporting of experiments followed the ARRIVE guidelines. Mice were housed in the Laboratory Animal Resources and Research (LARR) facility under a 12-h light: 12-h dark cycle. *Pgr*-Cre mice were generated previously [31]. *Ezh2^flox/flox^* mice (# 022616) and *Pten^flox/flox^* mice (# 006440) were purchased from the Jackson Laboratory.

### 2.2. Genotyping and DNA Recombination Analysis

Genotyping was conducted using genomic DNA isolated from mouse tails. Recombination of the *Ezh2* and *Pten* conditional alleles at the DNA level was analyzed using uterine DNA samples. Primer information for *Ezh2* and *Pten* was obtained from the Jackson Laboratory. Gene-specific primers were used to detect *Ezh2* (5′-CATGTG-CAGCTTTCTGTTCA-3′ and 5′-CACAGCCTTTCTGCTCACTG-3′; wild-type band = 203 bp and flox band = ~300 bp), *Pten* (5′-CAAGCACTCTGCGAACTGAG-3′ and 5′-AAGTTTTTGAAGGCAAGATGC-3′; wild-type band = 156 bp and flox band = 328 bp), *Ezh2* recombination (5′-CCCATGTTTAAGGGCATAGTGACATG-3′ and 5′-TCGAGGGACCTAATAACTCGTATAGCA-3′) [32], and *Pten* recombination (5′-ACTCAAGGCAGGGATGAGC-3′ and 5′-AATCTAGGGCCTCTTGTGCC-3′) [33].

### 2.3. Histology, Immunohistochemistry, and Immunofluorescence

Uterine samples were fixed in 10% (*v*/*v*) neutral-buffered formalin (MilliporeSigma, Burlington, MA, USA), embedded in paraffin, and processed using the Texas A&M College of Veterinary Medicine & Biomedical Sciences Core Histology Laboratory. Sections (5 µm) were subjected to hematoxylin and eosin (H.E.) staining and Periodic Acid Schiff (PAS) staining to determine the histopathological features of the uterus/endometrial cancer. Immunohistochemistry and immunofluorescence procedures were detailed elsewhere [34]. Briefly, slides were deparaffinized, rehydrated, and boiled in sodium citrate buffer (pH = 6) to restore antigenicity. Sections were then blocked and incubated sequentially with primary antibodies (Table 1) overnight at 4 °C and biotinylated secondary antibodies (immunohistochemistry) or fluorescent secondary antibodies (immunofluorescence). For immunohistochemistry, avidin-biotin complex (# PK-6100; Vector Laboratories, Burlingame, CA, USA) and NovaRed (#SK-4800; Vector Laboratories) were used to amplify the signal and develop the slides, respectively. For immunohistochemistry, slides were mounted with Fisher mounting medium. In contrast, slides from immunofluorescence experiment were directly mounted using DAPI-containing medium to counterstain the nuclei.

### 2.4. Western Blot

Western blot was performed as described elsewhere [34]. Uterine tissue homogenates were prepared from mice at 14 days of age, and 30 µg of protein samples were subject to electrophoresis. Incubation of primary antibodies (Table 1) was carried out overnight at 4 °C. Western blot images were quantified using NIH Image J (version 1.52p, Bethesda, MD, USA).

### 2.5. Hypoxia Staining

Hypoxia staining was performed using Hypoxyprobe Plus Kit (# HP2-100Kit, Hypoxyprobe, Burlington, MA, USA) based on the manufacturer’s protocol. Briefly, hypoxyprobe-1 (pimonidazole) was administered prior to sample collection. Slides were incubated at 60 °C for 20 min, deparaffinized, and then rehydrated. The slides were then treated with H_2_O_2_ to inactivate endogenous peroxidase activity. The antigenicity was restored by boiling the sides in sodium citrate buffer (pH = 6). After being blocked with non-immune sera, slides were incubated with FITC-MAb1 (1:50) at 4 °C overnight. In hypoxic cells/tissues, pimonidazole is bioreductively activated to form stable adducts (PIM) detectable by immunostaining. The next day, slides were washed and then mounted with DAPI-containing medium and examined under a fluorescence microscope (Olympus, Waltham, MA, USA).

### 2.6. Enzyme-Linked Immunosorbent Assay (ELISA)

The levels of mouse neutrophil elastase/ELA2 or tumor necrosis factor α (TNFα) in the serum/uterine tissue homogenates were measured using Quantikine ELISA kit (R&D, Minneapolis, MN, USA) based on manufacturer’s instructions. Serum samples were diluted (1:5–1:100) to meet the detection range, with at least three biological replicates per experimental group and two technical replicates per sample. Tissue homogenates were prepared and then treated with repeated freezing and thawing cycles. The optical density (OD) values were measured using a microplate reader (Bio-Rad, Hercules, CA, USA) at dual wavelengths (450 nm and 540 nm). OD values were corrected by subtracting readings at 540 nm from those at 450 nm. The concentration of ELA2 or TNFα was calculated using an online tool (http://elisaanalysis.com (accessed on 28 January 2020)).

### 2.7. Hormone Assays

Serum estradiol and progesterone levels from nine-week-old *Pten^d/d^* and *Pten^d/d^; Ezh2^d/d^* mice were determined using the Ligand Assay and Analysis Core (Center for Research in Reproduction, University of Virginia). Assay details are available at https://med.virginia.edu/research-in-reproduction/ligand-assay-analysis-core/ (accessed on 2 July 2021).

### 2.8. Quantitative Reverse Transcription-PCR

Total RNA was isolated from uterine tissues using RNeasy Mini Kit (Qiagen, Germantown, MD, USA). Reverse transcription (RT) was performed using a SuperScript III reverse transcriptase (ThermoFisher Scientific, Waltham, MA, USA) in the presence of 500 ng total RNA. Quantitative RT-PCR (qRT-PCR) was conducted using gene-specific primers as described [34]. At least three biological replicates per group and two technical replicates per sample were included. Relative levels of gene expression were determined as described elsewhere [35], with ribosomal protein L19 (*Rpl19*) as an internal control. Primers include cellular retinoic acid binding protein II (*Crabp2*) (5′-ATGCCTAACTTTTCTGGCAACT-3′ and 5′-GCACAGTGGTGGAGGTTTTGA-3′; PrimerBank ID 33469075a1), dehydrogenase/reductase (SDR family) member 9 (*Dhrs9*) (5′-ATGCTGTTTTGGTTGTTGGCT-3′ and 5′-GTTCTGGCTGCTAAGTTTCCA-3′; PrimerBank ID 30425272a1), *Ezh2^del^*, *Pten*, keratin 14 (*Krt14*), Δ*Np63*, chemokine (C-X-C motif) ligand 5 (*Cxcl5*), chemokine (C-X-C motif) receptor 2 (*Cxcr2*), and aldehyde dehydrogenase 3 family, member B2 (*Aldh3b2*) [30,33,36,37].

### 2.9. Statistical Analysis

Statistical analysis was conducted using GraphPad Prism 9 (San Diego, CA, USA). Unpaired two-tailed *t*-test was used to compare means between two groups. One-way analysis of variance (ANOVA) and Tukey’s multiple comparison test were used to compare means among multiple groups. Kaplan–Meier survival curves were analyzed using Log-rank (Mantel-Cox) test. Data are means ± s.e.m. A *p* value of less than 0.05 was reported as statistically significant (* *p* < 0.05, ** *p* < 0.01, *** *p* < 0.001, and **** *p* < 0.0001).

## 3. Results

### 3.1. Generation of Mice with Conditional Deletion of Ezh2 and Pten

**Figure 1:**
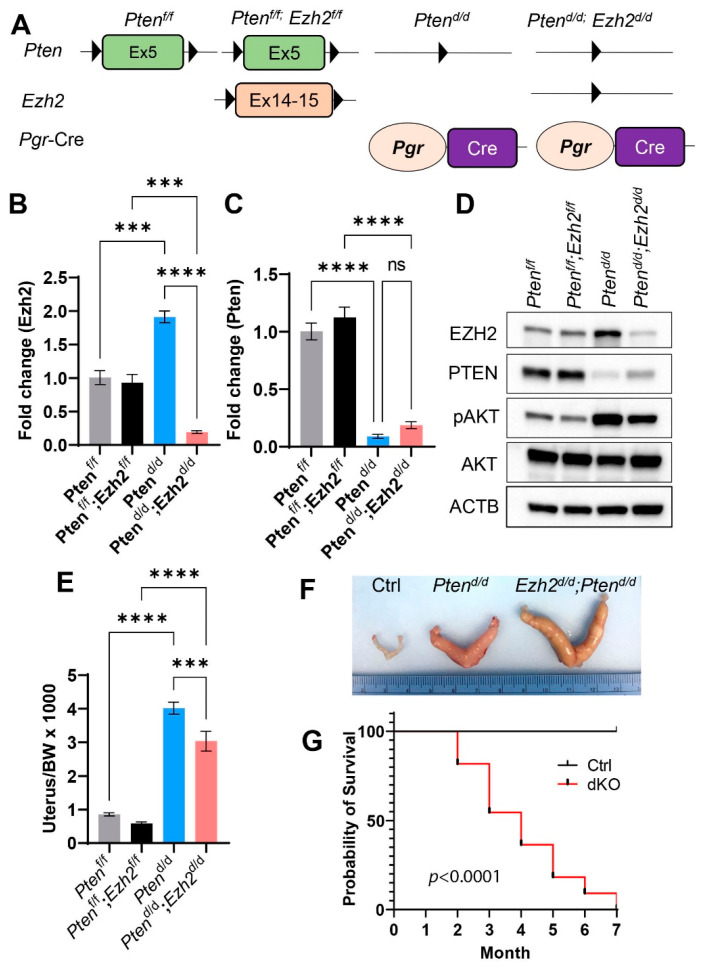
Generation of mice with conditional deletion of *Pten* and *Ezh2* in the uterus. (**A**) A schematic of Cre-LoxP approach to generate conditionally deleted mice. (**B**,**C**) Transcript levels of *Ezh2* and *Pten* in the mouse uterus at 14 days of age. *n* = 3–4. Data are mean ± s.e.m. *** *p* < 0.001 and **** *p* < 0.0001. ns, not significant. (**D**) Western blot analysis of EZH2, PTEN, pAKT, and AKT in 14-day-old *Pten^f/f^, Pten^f/f^; Ezh2^f/f^*, *Pten^d/d^*, and *Pten^d/d^; Ezh2^d/d^* uteri. ACTB was used as an internal control. *n* = 4. (**E**) Ratios of the uterus/body weight (BW) in *Pten^f/f^, Pten^f/f^; Ezh2^f/f^*, *Pten^d/d^*, and *Pten^d/d^; Ezh2^d/d^* mice at three weeks of age. *n* = 9–13. Data are mean ± s.e.m. *** *p* < 0.001 and **** *p* < 0.0001. (**F**) Gross image of uteri from nine-week-old *Pten^f/f^* (Ctrl), *Pten^d/d^*, and *Pten^d/d^; Ezh2^d/d^* mice. (**G**) Kaplan–Meier survival curves of *Pten^f/f^; Ezh2^f/f^* (Ctrl) and *Pten^d/d^; Ezh2^d/d^* mice. *n* = 11.

Conditional ablation of PTEN using *Pgr*-Cre (*Pten^d/d^*) in the mouse uterus leads to the development of endometrial cancer [20,33]. *Ezh2* conditional knockout mice are free of endometrial cancer but develop stratified uterine epithelia that contain basal-like cells absent in the normal uterus [30]. To determine the role of EZH2 in endometrial cancer development, we created mice with simultaneous deletion of *Ezh2* and *Pten* (*Pten^d/d^; Ezh2^d/d^*) or *Pten* only (*Pten^d/d^*) in the uterus using Cre-LoxP approach (Figure 1A). *Pten^f/f^* and *Pten^f/f^; Ezh2^f/f^* mice were included as controls. Recombination of *Pten* and *Ezh2* alleles occurred specifically in the uteri of *Pten^d/d^* and *Pten^d/d^; Ezh2^d/d^* mice but not controls (Figure S1A). Conditional deletion of *Ezh2* and *Pten* at the transcript levels was demonstrated using qRT-PCR (Figure 1B,C). We also verified the ablation of PTEN and EZH2 proteins by western blot (Figure 1D) and immunostaining (Figure S1B–G). Of note, expression of EZH2 was increased in *Pten^d/d^* uteri compared with age-matched controls (i.e., *Pten^f/f^* and *Pten^f/f^; Ezh2^f/f^*) (Figure 1D). Loss of PTEN was expected to enhance PI3K/AKT activity, as PTEN inhibits PI3K-AKT pathway [38]. Indeed, increased expression of phospho-AKT (pAKT) was found in the uteri of *Pten^d/d^* and *Pten^d/d^; Ezh2^d/d^* mice versus controls (Figure 1D and Figure S1H–J).

EZH2 is a histone methyltransferase that methylates H3K27 [23]. To determine if loss of EZH2 affected H3K27me3 levels, we examined the expression of H3K27me3 using uteri from controls, *Pten^d/d^*, and *Pten^d/d^; Ezh2^d/d^* mice at 14 days of age. Immunostaining revealed reduced levels of H3K27me3 in *Pten^d/d^; Ezh2^d/d^* uteri compared with *Pten^d/d^* uteri (Figure S1K–M). Although EZH2 expression was increased in *Pten^d/d^* uteri (Figure 1D), H3K27me3 levels were not altered in *Pten^d/d^* uteri in comparison with the control (Figure S1N,O). The above results indicate successful ablation of EZH2 and PTEN in the mouse uterus.

### 3.2. Loss of EZH2 Reduces Tumor Burden during Early Carcinogenesis but Negatively Impacts Disease Outcome

Consistent with the documented epithelial hyperplasia and endometrial cancer development resulting from loss of PTEN [20,39], the uterine weights of *Pten^d/d^* and *Pten^d/d^; Ezh2^d/d^* mice were significantly increased compared with controls at three weeks of age (Figure 1E). Simultaneous loss of EZH2 and PTEN reduced the uterine weight compared with *Pten^d/d^* mice (Figure 1E). However, the uteri of *Pten^d/d^; Ezh2^d/d^* mice were larger than those of *Pten^d/d^* mice at the age of nine weeks (Figure 1F). To assess the outcome of endometrial cancer in mice with conditional deletion of *Pten* and *Ezh2*, we generated Kaplan–Meier survival curves, which showed that *Pten^d/d^; Ezh2^d/d^* mice succumbed to death starting around two months of age (Figure 1G). As endometrial cancer in *Pten^d/d^* mice does not substantially affect the viability up through five months of age [20,33], current results suggest that deletion of *Ezh2* negatively impacts the disease outcome.

**Figure 2:**
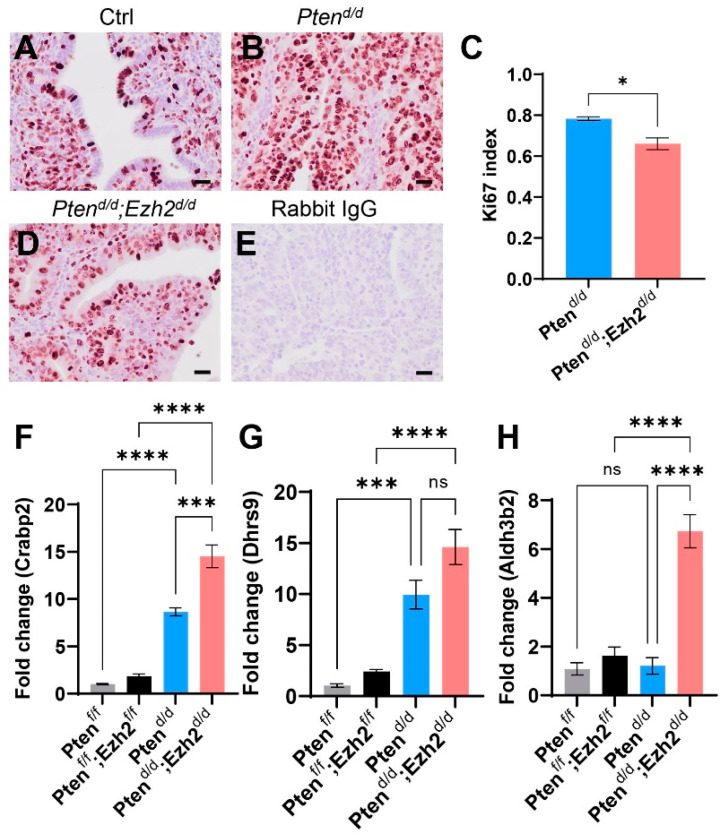
Reduced Ki67 index in the uteri of *Pten^d/d^; Ezh2^d/d^* mice compared with *Pten^d/d^* mice. (**A**–**E**) Immunohistochemical analysis of Ki67 using three-week-old *Pten^f/f^* (Ctrl), *Pten^d/d^*, and *Pten^d/d^; Ezh2^d/d^* mice. Ki67 index for *Pten^d/d^* and *Pten^d/d^; Ezh2^d/d^* mice is shown in panel (**C**). *n* = 3. Data are mean ± s.e.m. * *p* < 0.05. Scale bar = 20 µm (**A**,**B**,**D**,**E**). (**F**–**H**) Transcript levels of *Crabp2*, *Dhrs9*, and *Aldh3b2* in uterine tissues from *Pten^f/f^, Pten^f/f^; Ezh2^f/f^*, *Pten^d/d^*, and *Pten^d/d^; Ezh2^d/d^* mice at three weeks of age. *n* = 4. Data are mean ± s.e.m. *** *p* < 0.001 and **** *p* < 0.0001. ns, not significant.

As *Pten^d/d^; Ezh2^d/d^* mice showed a reduction in uterine weight compared with *Pten^d/d^* mice during early tumor development (Figure 1E), we sought to determine the effect of *Ezh2* deletion on the proliferation of endometrial cancer cells. Immunostaining of Ki67, a cell proliferation marker, was performed using uteri from three-week-old control, *Pten^d/d^*, and *Pten^d/d^; Ezh2^d/d^* mice. Results showed reduced Ki67 index (i.e., number of Ki67-positive cells/number of total cells) in *Pten^d/d^; Ezh2^d/d^* uteri versus *Pten^d/d^* uteri (Figure 2A–E). It has been reported that retinoic acid (RA) signaling inhibits endometrial cancer cell proliferation [40]. Herein, we found that several genes associated with RA synthesis were upregulated in *Pten^d/d^; Ezh2^d/d^* uteri versus *Pten^d/d^* uteri (Figure 2F–H). These genes encode DHRS9 that is involved in RA biosynthesis from retinaldehyde, CRABP2 that transports RA to the RA receptor, and ALDH3B2, an enzyme of the aldehyde dehydrogenase superfamily. The finding that deletion of *Ezh2* in *Pten^d/d^* uteri reduced endometrial cell proliferation during early carcinogenesis suggests an oncogenic role of EZH2.

### 3.3. Ezh2 and Pten Deletion Enhances the Accumulation of Intraluminal Neutrophils Compared with Pten Deletion Alone

**Figure 3:**
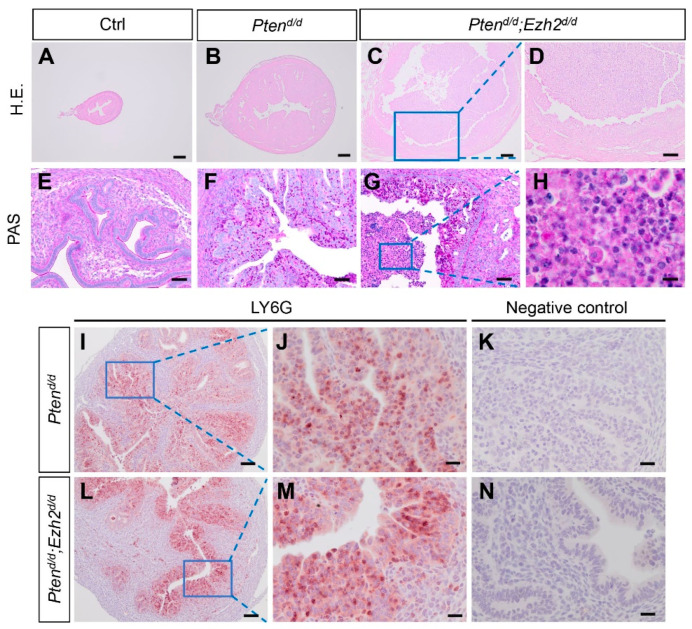
Deletion of *Ezh2* promotes accumulation of intraluminal neutrophils in PTEN-inactivated uteri. (**A**–**H**) Histological analyses of uteri from 1-month-old *Pten^f/f^, Pten^d/d^*, and *Pten^d/d^; Ezh2^d/d^* mice using H.E. staining (**A**–**D**) and PAS staining (**E**–**H**). Panels (**D**,**H**) are high power images of the boxed areas of panels (**C**,**G**), respectively. (**I**–**N**) Immunostaining of LY6G using uteri from three-week-old *Pten^d/d^* and *Pten^d/d^; Ezh2^d/d^* mice. Panels (**J**,**M**) are high power images of the boxed areas of panels (**I**,**L**), respectively. Panels (**K**,**N**) are negative controls where the primary antibody was replaced by isotype-matched IgG. At least three independent samples per genotype were examined. Scale bar = 10 µm (**H**), 20 µm (**J**,**K**,**M**,**N**), 50 µm (**E**–**G**), 100 µm (**D**,**I**,**L**), and 200 µm (**A**–**C**).

To begin to understand the cellular basis of altered endometrial cancer progression in *Pten^d/d^; Ezh2^d/d^* mice, we examined the morphological/histological changes of the uterus. At one month of age, H.E. staining showed that the size of the uteri was enlarged in both *Pten^d/d^* and *Pten^d/d^; Ezh2^d/d^* mice compared with controls (Figure 3A–D). Of note, some *Pten^d/d^; Ezh2^d/d^* mice showed a ring-like uterine lumen on the cross section, with loss of uterine epithelia and massive accumulation of polymorphonuclear neutrophils and cell debris (Figure 3C,D,G,H and Figure S2).

Neutrophils are critical for cancer development and metastasis [41]. To determine a timeline of the observed intraluminal neutrophil accumulation, tumor development was next examined in *Pten^d/d^; Ezh2^d/d^* mice at three weeks of age. To better visualize neutrophil infiltration, we performed immunostaining of lymphocyte antigen 6 complex, locus G (LY6G), a neutrophil marker. Although neutrophil infiltration occurred in both *Pten^d/d^; Ezh2^d/d^* and *Pten^d/d^* mice, no substantial accumulation of intraluminal neutrophils was observed in *Pten^d/d^; Ezh2^d/d^* or *Pten^d/d^* mice at this stage (Figure 3I–N). Negative controls are shown in Figure 3K and N. CXCL5 and CXCR2 are critical for recruiting neutrophils to endometrial cancer lesions [39]. To determine whether expression of *Cxcl5* and *Cxcr2* by uterine epithelial cells was altered upon *Ezh2* deletion, we isolated uterine epithelia from *Pten^d/d^* and *Pten^d/d^; Ezh2^d/d^* mice. Results showed that the expression levels of *Cxcl5* and *Cxcr2* were not statistically different between *Pten^d/d^; Ezh2^d/d^* and *Pten^d/d^* mice, despite a drastic reduction of *Ezh2* expression in the epithelia from *Pten^d/d^; Ezh2^d/d^* mice (Figure S3).

**Figure 4:**
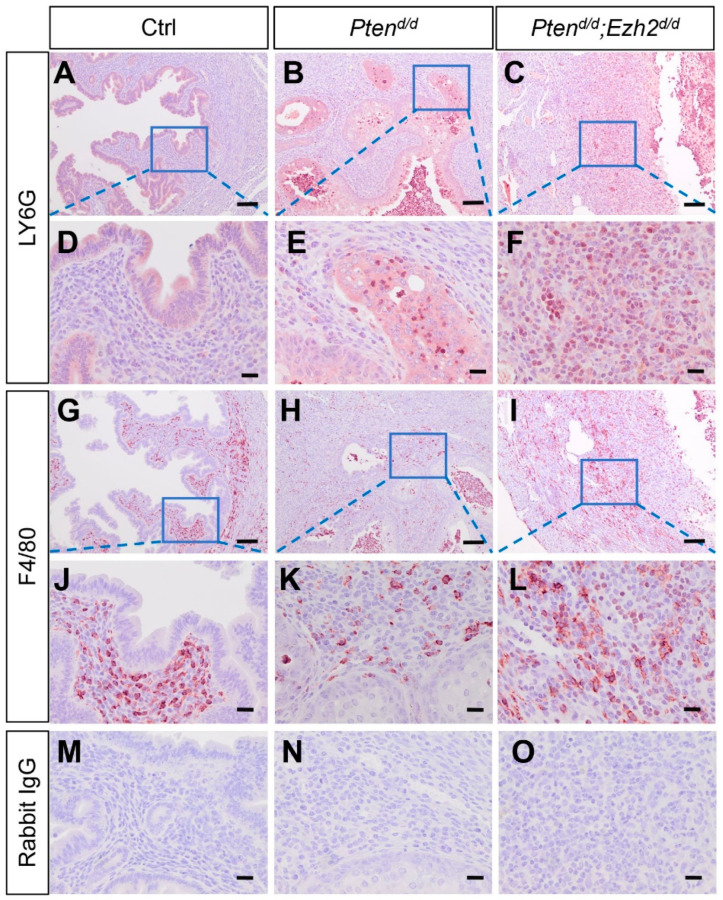
Deletion of *Ezh2* in PTEN-inactivated uteri causes heightened inflammation. (**A**–**L**) Immunostaining of LY6G and F4/80 in the uteri from nine-week-old *Pten^f/f^*, *Pten^d/d^*, and *Pten^d/d^; Ezh2^d/d^* mice. Panels (**D**–**F**,**J**–**L**) are high power images of the boxed areas of panels (**A**–**C**,**G**–**I**), respectively. (**M**–**O**) Negative controls where the primary antibody was replaced with isotype–matched IgG. At least three independent mice per group were examined. Scale bar = 100 µm (**A**–**C**,**G**–**I**) and 20 µm (**D**–**F**,**J**–**O**).

To further assess the extent of neutrophil accumulation during tumor progression, we examined the uteri of *Pten^d/d^; Ezh2^d/d^* and *Pten^d/d^* mice at nine weeks of age. While LY6G-positive neutrophils were sparse in control uteri (Figure 4A,D), they were increased within the epithelia in *Pten^d/d^* mice (Figure 4B,E). Strikingly, abundant neutrophils were found in *Pten^d/d^; Ezh2^d/d^* uteri encompassing both the stroma and epithelia, despite a substantial loss of uterine epithelia in these mice (Figure 4C,F and Figure S4). In addition, all *Pten^d/d^; Ezh2^d/d^* mice developed the aforementioned ring-like uterine lumen when cross sections were examined (Figure S4C). As macrophage is involved in the clearance of cellular debris, we also examined the presence of macrophage by immunostaining of F4/80. Results showed that immunoreactive signals of F4/80 were mainly localized to the stroma of control and *Pten^d/d^* uteri (Figure 4G,H,J,K). However, F4/80-positive macrophages were accumulated in both uterine epithelia and stroma of *Pten^d/d^; Ezh2^d/d^* mice (Figure 4I,L). Negative controls are shown in Figure 4M–O.

**Figure 5:**
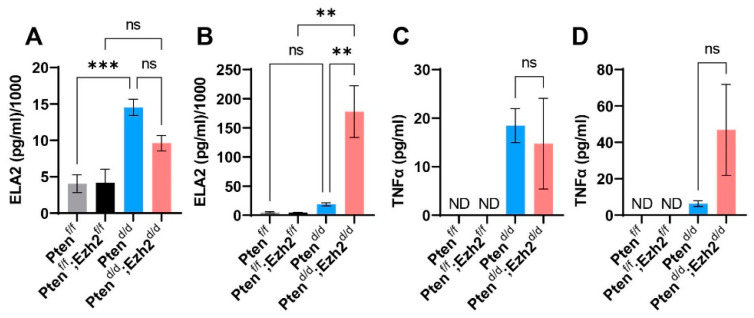
Analysis of ELA2 and TNFα levels using ELISA. (**A**,**B**) Serum levels of ELA2 at the age of one month (**A**) and nine weeks (**B**). (**C**,**D**) TNFα levels in uterine tissues at the age of one month (**C**) and in the serum at nine weeks (**D**). *n* = 3–4. Data are mean ± s.e.m. ** *p* < 0.01 and *** *p* < 0.001. ns, not significant. ND, not detected.

To determine a potential link between the stage-dependent intraluminal accumulation of neutrophils and chronic inflammation in *Pten^d/d^; Ezh2^d/d^* mice, we measured the levels of serum ELA2, a serine proteinase produced by neutrophils during inflammation [42]. It was found that ELA2 levels were not statistically different between *Pten^d/d^; Ezh2^d/d^* and *Pten^d/d^* mice at the age of 1 month (Figure 5A). In contrast, serum ELA2 levels were markedly elevated in *Pten^d/d^; Ezh2^d/d^* mice at nine weeks of age, compared with age-matched *Pten^d/d^* mice and controls (Figure 5B). In addition, we determined the levels of another important pro-inflammatory cytokine, TNFα, in uterine tissue homogenates or the serum of control, *Pten^d/d^*, and *Pten^d/d^; Ezh2^d/d^* mice. The levels of TNFα were below the limit of detection in controls. However, TNFα levels were elevated in *Pten^d/d^* and *Pten^d/d^; Ezh2^d/d^* mice, although a statistical significance between *Pten^d/d^* and *Pten^d/d^; Ezh2^d/d^* mice was not achieved due to sample variations (Figure 5C,D). Collectively, loss of EZH2 enhanced the accumulation of intraluminal neutrophils, leading to heightened chronic inflammation.

### 3.4. Factors Contributing to the Developmental Trajectory of Endometrial Cancer Lacking PTEN and EZH2

**Figure 6:**
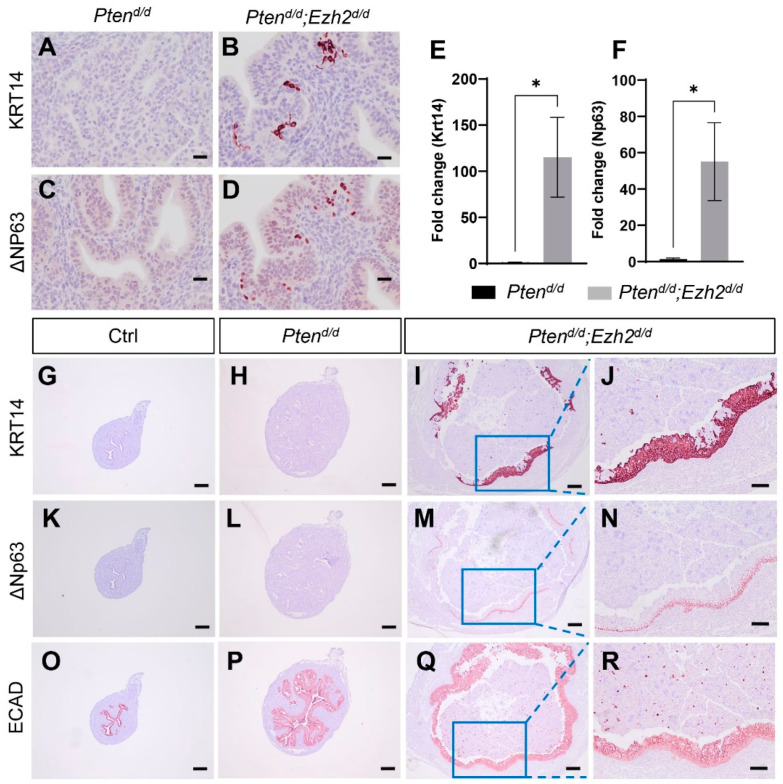
Deletion of *Ezh2* intensifies epithelial stratification in PTEN-depleted uteri. (**A**–**D**) Immunostaining of KRT14 and ΔNP63 using uteri from three-week-old *Pten^d/d^* and *Pten^d/d^; Ezh2^d/d^* mice. (**E**,**F**) Transcript levels of *Krt14* and Δ*Np63* in uterine epithelia isolated from three-week-old *Pten^d/d^* and *Pten^d/d^; Ezh2^d/d^* mice. *n* = 4–5. Data are mean ± s.e.m. * *p* < 0.05. Note that one sample from the *Pten^d/d^* group had undetectable Δ*Np63* expression and was not included in panel (**F**). (**G**–**R**) Immunostaining of KRT14 (**G**–**J**), ΔNP63 (**K**–**N**), and ECAD (**O**–**R**) in uteri from one-month-old *Pten^f/f^* (Ctrl), *Pten^d/d^*, and *Pten^d/d^; Ezh2^d/d^* mice. Panels (**J**,**N**,**R**) represent high power images of the boxed areas of panels (**I**,**M**,**Q**), respectively. At least three independent samples were examined for each genotype. Scale bar = 20 µm (**A**–**D**), 100 µm (**J**,**N**,**R**), and 200 µm (**G**–**I**,**K**–**M**,**O**–**Q**).

As our previous studies showed that conditional loss of EZH2 in the uterus elicits epithelial stratification [30], we asked the question of whether uterine epithelial stratification occurred in *Pten^d/d^; Ezh2^d/d^* mice during tumor development. To approach this question, we first performed immunostaining of KRT14 and ΔNp63, two basal cell markers. Immunoreactive signals for both KRT14 and ΔNp63 were detectable as early as three weeks of age in *Pten^d/d^; Ezh2^d/d^* mice, but not in age-matched *Pten^d/d^* mice and controls (Figure 6A–D). Supporting the immunohistochemical results, transcript levels of *Krt14* and Δ*Np63* were increased in uterine epithelia of *Pten^d/d^; Ezh2^d/d^* mice compared with *Pten^d/d^* mice (Figure 6E,F). Further, *Pten^d/d^; Ezh2^d/d^* mice contained stratified epithelia positively stained for KRT14 and ΔNp63 at the age of one month (Figure 6I,J,M,N and Figure S2). In the severe case with massive accumulation of intraluminal neutrophils, nearly the entire uterine lumen was surrounded by stratified epithelia (Figure 6I,M and Figure S5), in sharp contrast to age-matched *Pten^d/d^* and control mice, where minor to negligible staining of KRT14 and ΔNp63 was found (Figure 6G,H,K,L). Immunostaining of ECAD was conducted to show the epithelial components of the uterus (Figure 6O–R). Thus, conditional deletion of *Ezh2* in *Pten^d/d^* uteri exacerbated uterine epithelial stratification that might negatively impact the disease outcome.

**Figure 7:**
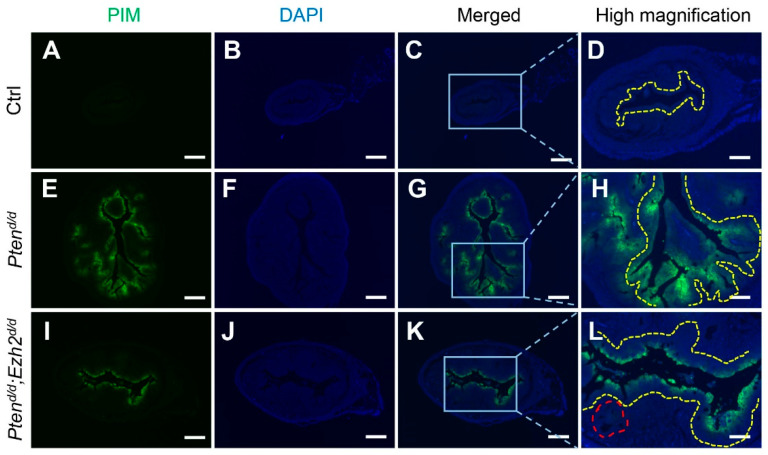
Reduced hypoxia in the uteri of *Pten^d/d^; Ezh2^d/d^* mice compared with *Pten^d/d^* mice. (**A**–**L**) Immunofluorescence staining of pimonidazole protein adducts using one-month-old *Pten^f/f^* (Ctrl), *Pten^d/d^*, and *Pten^d/d^; Ezh2^d/d^* uteri. PIM, pimonidazole protein adducts. Panels (**D**,**H**,**L**) are high power images of the boxed areas of panels (**C**,**G**,**K**), respectively. Yellow dashed lines denote the luminal epithelial component or epithelia adjacent to the uterine lumen. Note the reduced extent of epithelial hypoxia and diminished hypoxia in the glandular-like component (red dashed line) in *Pten^d/d^; Ezh2^d/d^* uteri. At least three independent mice were examined for each genotype. DAPI was used to counterstain the nuclei. Scale bar = 250 µm (**A**–**C**,**E**–**G**,**I**–**K**) and 100 µm (**D**,**H**,**L**).

Hypoxia is implicated in endometrial cancer development [39,43]. We found that hypoxia signals were mainly localized to the epithelial compartment of the uteri from *Pten^d/d^* and *Pten^d/d^; Ezh2^d/d^* mice at one month of age (Figure 7E–L), with background levels of staining in the control uteri (Figure 7A–D). Interestingly, reduced hypoxia was observed in *Pten^d/d^; Ezh2^d/d^* uteri compared with *Pten^d/d^* uteri (Figure 7E–L). As relieving tumor hypoxia enhances the tumoricidal activity of neutrophils in *Pten^d/d^* mouse model [44], lower hypoxic levels in the *Pten^d/d^; Ezh2^d/d^* uteri may facilitate the debridement of cancer epithelia by neutrophils, resulting in increased intraluminal accumulation of cancer cells/debris.

**Figure 8:**
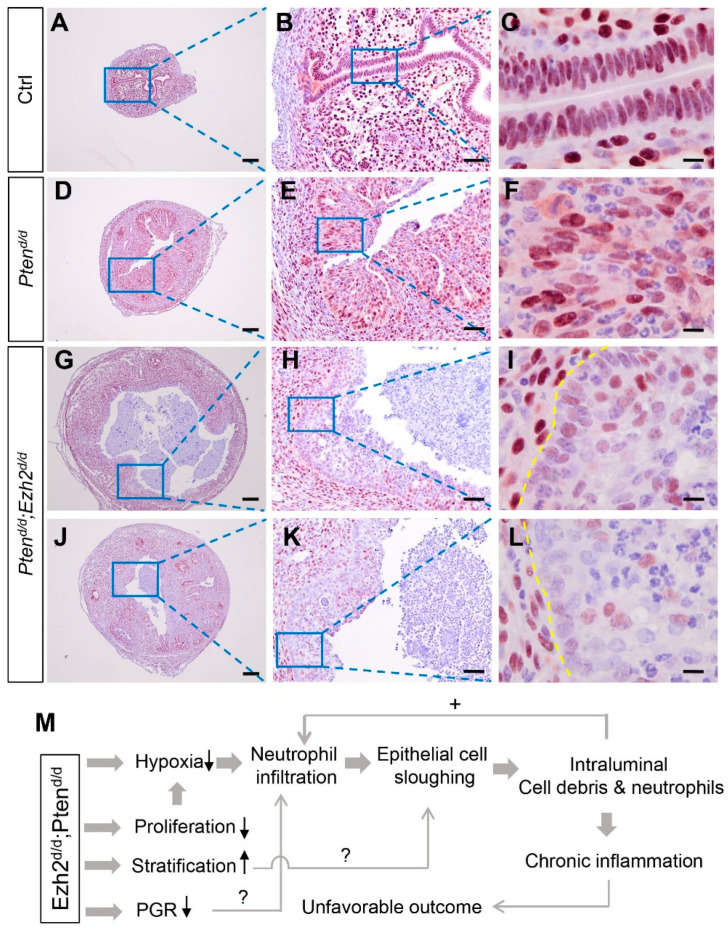
Expression of PGR in *Pten^d/d^* and *Pten^d/d^; Ezh2^d/d^* uteri. (**A**–**L**) Immunostaining of PGR in the uteri of 1-month-old *Pten^f/f^* (Ctrl), *Pten^d/d^*, and *Pten^d/d^; Ezh2^d/d^* mice. Panels (**G**–**I**) and Panels (**J**–**L**) represent two independent *Pten^d/d^; Ezh2^d/d^* mice with different disease progression. Panels (**B**,**E**,**H**,**K**) are high power images of the boxed areas of panels (**A**,**D**,**G**,**J**), respectively. Panels (**C**,**F**,**I**,**L**) are high power images of the boxed areas of panels (**B**,**E**,**H**,**K**), respectively. At least three independent samples were examined for each genotype. Scale bar = 10 µm (**C**,**F**,**I**,**L**), 50 µm (**B**,**E**,**H**,**K**), and 200 µm (**A**,**D**,**G**,**J**). Yellow dashed lines demarcate the border between uterine stroma and epithelia. (**M**) A hypothetical model of endometrial cancer development in mice harboring uterine deletion of both *Pten* and *Ezh2*.

Progesterone receptor signaling plays important roles in endometrial cancer development, and loss of PGR is linked to the development of aggressive endometrial cancer [45,46]. Immunostaining was performed to examine whether PGR expression was altered in *Pten^d/d^; Ezh2^d/d^* uteri. Results showed reduced PGR expression in the luminal epithelia of 1-month-old *Pten^d/d^; Ezh2^d/d^* mice (Figure 8G–L) compared with age-matched *Pten^d/d^* mice and controls (Figure 8A–F). Hormone assays showed that the levels of estrogen and progesterone were comparable between *Pten^d/d^; Ezh2^d/d^* mice and *Pten^d/d^* mice (Figure S6), indicating that ablation of EZH2 did not affect the levels of ovarian steroid hormones. As PGR signaling interacts with estrogen signaling that promotes neutrophil recruitment [47,48], reduced PGR expression may alter estrogen action and inflammation. Collectively, these studies identified potential contributing factors to the unfavorable outcome of endometrial cancer lacking both PTEN and EZH2 (Figure 8M).

## 4. Discussion

Both *PTEN* and *EZH2* play important roles in endometrial cancer. The mutation of *PTEN* gene has been identified in ~20% of human endometrial hyperplasia, suggesting its importance in early cancer development [49]. The frequency of *PTEN* mutation appears to be associated with the histotypes of endometrial cancer, as *PTEN* mutation occurs in ~40% of endometrioid cancers but only 5% of serous or clear cell endometrial cancers [19]. EZH2 is overexpressed in endometrial cancer, and its downregulation in endometrial cancer cells inhibits cell proliferation [24,50]. A more recent study has identified a correlation between overexpression of EZH2 in endometrial cancer patients and disease-free and overall survival [51]. This report has further demonstrated that silencing *EZH2* in endometrial cancer cells impairs the expression of growth-related genes such as peroxiredoxin 6 (*PRDX6*) [51]. The mechanisms underlying EZH2 action in endometrial cancer progression remain incompletely understood. However, it appears that microRNA-361/Twist axis plays an important role in mediating the role of EZH2 in driving endometrial cancer development [52]. The evidence points to the therapeutic potential of targeting EZH2. However, EZH2 may also function as a tumor suppressor in myeloma and pancreatic tumor [53,54]. It has been shown that loss of EZH2 in the mouse uterus enhances epithelial cell proliferation [55,56,57] and induces epithelial stratification [30]. Herein, we found that conditional deletion of both *Ezh2* and *Pten* reduced cell proliferation and uterine growth during early carcinogenesis but exacerbated intraluminal neutrophil accumulation and chronic inflammation during tumor progression, leading to an unfavorable disease outcome. Current results revealed dual roles of EZH2 in the development of endometrial cancer lacking *Pten*, a gene frequently mutated in endometrioid carcinomas.

The uterine weights of *Pten^d/d^; Ezh2^d/d^* mice were lower than those of *Pten^d/d^* mice at three weeks of age, accompanied by reduced cell proliferation revealed by Ki67-staining. As EZH2 inhibits uterine epithelial cell proliferation and uterine growth [30,55,56,57], our results suggest that EZH2 plays distinct roles in normal uterine epithelial cells versus malignant epithelial cells. Supporting the assumption that the role of EZH2 in PTEN-depleted epithelial cells differs from that in PTEN-expressing epithelial cells, it was reported that loss of PTEN or activation of AKT switches the tumor suppressive role of EZH2 to an oncogenic function [58]. Interestingly, AKT activation is also implicated in normal and estrogen-induced uterine epithelial cell proliferation [59]. These findings support a complex, yet contextually dependent, role of EZH2 in cancer development.

Neutrophils are the first-line defenders that actively participate in host defense, tissue damage, and inflammatory disease [60]. Tumor-associated neutrophils play important roles in tumor microenvironment, where N1 neutrophils are anti-tumorigenic and N2 neutrophils are pro-tumorigenic [41,61]. The pro-tumorigenic action of neutrophils is generally associated with their effects on cancer cell invasion, extracellular matrix remodeling, and angiogenesis [61]. Although the oncogenic role of EZH2 has been documented, some in vivo experiments suggest a tumor-suppressive function of EZH2. One study showed that loss of EZH2 promotes KRas^G12D^-driven oncogenesis in pancreatic cancer [54]. In another report, deletion of *Ezh2* accelerates Kras-driven lung adenocarcinoma in a mouse model [62]. In both cases, EZH2 appears to play a role in controlling inflammatory microenvironment [54,62]. In the present study, we found that tumor burden was reduced in *Pten^d/d^; Ezh2^d/d^* mice during early tumor development, revealing an oncogenic role of EZH2 in endometrial cancer development. However, unfavorable cancer outcomes were observed in these mice compared with *Pten^d/d^* mice. The latter effect is likely non-cell autonomous, as dysregulation of EZH2 in cancer cells is known to alter immune response [63]. Indeed, massive accumulation of intraluminal neutrophils is a hallmark of the endometrial cancer in *Pten^d/d^; Ezh2^d/d^* mice at nine weeks of age. Our finding is also consistent with a previous report that increased levels of intratumoral neutrophils correlate with a poor cancer outcome [64].

The underlying mechanisms that promote the heightened inflammation in *Pten^d/d^; Ezh2^d/d^* mice remain unclear. However, several important contributing factors were identified by the present study. First, we found reduced hypoxia in the uteri of *Pten^d/d^; Ezh2^d/d^* mice at 1 month of age. Elegant studies have demonstrated that hypoxia increases neutrophil recruitment in endometrial cancer induced by PTEN depletion, which serves to restrain the development of endometrial cancer by debridement of the malignant cells [39,44]. Interestingly, reduction of hypoxia causes attenuated neutrophil infiltration. However, these neutrophils gain more efficient capability of attacking cancer cells [44]. Loss of EZH2 limited the extent of hypoxia in *Pten^d/d^; Ezh2^d/d^* mice, likely enhancing the tumoricidal effect of neutrophils [44]. Intraluminal accumulation of cancer cells/debris would in turn stimulate neutrophil influx and cause heightened immune reactions, forming a vicious cycle and resulting in chronic inflammation and/or eliciting secondary infectious event. The exact reasons of how EZH2 ablation led to reduced hypoxia is unclear. However, increased vascularization in *Pten^d/d^; Ezh2^d/d^* uteri (Fang X and Li Q, unpublished observation) may be one of the reasons. Second, conditional deletion of *Ezh2* potentiated epithelial stratification in *Pten^d/d^* mice. The uterus contains simple columnar epithelial cells expressing KRT8 but not KRT14 and p63 [65]. Current results showed that stratified epithelial markers KRT14 and ΔNp63 were expressed earlier in *Pten^d/d^; Ezh2^d/d^* uteri than *Pten^d/d^* uteri, consistent with our previous finding that loss of EZH2 in the uterus promotes the development of basal cells and stratified epithelia [30]. The intensified epithelial stratification in *Pten^d/d^; Ezh2^d/d^* uteri likely reflected the additive effect of loss of EZH2 and PTEN. Uterine epithelial stratification is a pathological event that alters the polarity and function of epithelial cells [66,67]. It is possible that epithelial stratification adversely impacts the progression of endometrial cancer due to altered epithelial cell properties. The role of epithelial stratification in endometrial cancer development in our model requires further investigation. Finally, it was found that epithelia adjacent to the uterine lumen had reduced expression of PGR in *Pten^d/d^; Ezh2^d/d^* mice at one month of age, when epithelial stratification intensified and marked accumulation of intraluminal neutrophils occurred. PGR loss has been associated with increased cell proliferation and metastasis [45,46]. PGR signaling antagonizes estrogen signaling during tumor development [68]. Estrogen is known to promote neutrophil recruitment during mammary involution or breast cancer development [47,48]. Thus, it is tempting to speculate that the reduction of PGR expression is associated with estrogen-directed neutrophil infiltration and heightened inflammation, which merits further investigation.

Endometrial cancer in *Pten^d/d^* mice is not metastatic to other organs even at 25–36 weeks of age [20,33,69]. However, dysregulation of several key regulators/signaling pathways may trigger metastasis. We have shown that conditional deletion of transforming growth factor β type 1 receptor (*Tgfbr1*) in *Pten^d/d^* mice promotes pulmonary metastases [33]. Lung metastasis was also reported in a mouse model where PTEN-ablated and K-ras expressed endometrial cancer cells were grafted [70]. In addition, conditional deletion of both *Pten* and dicer 1, ribonuclease type III (*Dicer1*) in the mouse uterus triggers adnexal metastasis [71]. EZH2 expression has been linked to endometrial cancer cell invasion and metastasis [50]. As mice conditionally overexpressing EZH2 are available [72], future investigations are needed to determine whether conditional overexpression of EZH2 in PTEN-depleted uteri impacts metastasis.

From a systems biology perspective, the functions of cells are achieved and coordinated by numerous genes/pathways within a highly interactive network [73]. Cancer may develop when perturbations of protein-protein interactions occur due to gene mutations [74]. Studies on protein-protein interaction networks in cancer may benefit cancer treatment by gaining a holistic view of mechanisms governing tumor development and discovering novel cancer drivers as well as therapeutic targets [75,76]. Recent studies have begun to explore protein-protein interaction networks in female reproductive cancers including endometrial cancer using an integrative computational approach [77]. Defining the interactome of endometrial cancer remains to be one of our key goals in the future.

The current study revealed dual roles of EZH2 in endometrial cancer development. We showed that ablation of EZH2 in the PTEN-inactivated endometrium reduced tumor burden during the early pathogenesis of endometrial cancer. However, these mice progressed to unfavorable disease condition, accompanied by intensified epithelial stratification, massive accumulation of intraluminal neutrophils, and heightened inflammation. These findings point to potentially unwanted effects of EZH2-targed therapy in cancer treatment. EZH2 has been implicated as an important cancer target in different types of cancers. Several EZH2 inhibitors have been developed and tested in clinical trials, such as GSK2816126 and tazemetostat [78,79]. No clinical trials of EZH2 inhibitors in endometrial cancer have been reported. The attenuation of early tumor growth upon *Ezh2* deletion in the current study suggests a therapeutic benefit by targeting EZH2 in endometrial cancer. However, the heightened inflammatory response and unfavorable disease outcome during tumor development strongly suggest that caution should be taken when designing anti-endometrial cancer strategies. Thus, stage-specific role of EZH2 should be considered. It is also tempting to postulate that a combination of EZH2 inhibitors and inflammatory modulators may be a possible approach to combat this most common gynecological disease.

## Figures and Tables

**Table 1:** Information of primary antibodies.

Name	Manufacturer	Cat. #	Species	IHC/IF	WB
EZH2	Cell Signaling (Danvers, MA, USA)	5246	Rabbit	1:400	1:1000
pAKT	Cell Signaling	4060	Rabbit	1:50	1:2000
Ki67	Cell Signaling	12202	Rabbit	1:500	
ECAD	Cell Signaling	3195	Rabbit	1:400	
KRT14	Thermo Fisher Scientific	PA5-16722	Rabbit	1:400	
ΔNp63	BioLegend (San Diego, CA, USA)	619001	Rabbit	1:200	
F4/80	Cell Signaling	70076	Rabbit	1:250	
LY6G	BioLegend	127601	Rat	1:500	
PTEN	Cell Signaling	9188	Rabbit		1:1000
AKT	Cell Signaling	4691	Rabbit		1:1000
H3K27me3	Cell Signaling	9733	Rabbit		1:1000
Histone H3	Cell Signaling	4499	Rabbit		1:5000
ACTB	MilliporeSigma	A3854	Mouse		1:50,000

IHC, immunohistochemistry; IF, immunofluorescence; WB, western blot.

## Data Availability

Data are included in the main article and supplementary figures.

## Supplementary Materials

The following supporting information can be downloaded at: https://www.mdpi.com/article/10.3390/cells11050909/s1. Figure S1: generation and validation of mice with conditional deletion of *Ezh2* and *Pten*; Figure S2: a summary of uterine histological features in *Pten^d/d^* and *Pten^d/d^; Ezh2^d/d^* mice at 1 month of age; Figure S3: levels of *Cxcl5*, *Cxcr2*, and *Ezh2* transcripts in uterine epithelial cells isolated from *Pten^d/d^* and *Pten^d/d^; Ezh2^d/d^* mice; Figure S4: intraluminal neutrophil infiltration in the uteri of *Pten^d/d^* and *Pten^d/d^; Ezh2^d/d^* mice; Figure S5: immunofluorescence of KRT14 and KRT8 using uteri from 1-month-old *Pten^d/d^; Ezh2^d/d^* mice; Figure S6: serum estrogen and progesterone levels in *Pten^d/d^* and *Ptend^/d^; Ezh2^d/d^* mice.

## References

[1] Siegel R.L., Miller K.D., Fuchs H.E., Jemal A. (2021). Cancer statistics, 2021. CA Cancer J. Clin..

[2] Di Cristofano A., Ellenson L.H. (2007). Endometrial carcinoma. Annu. Rev. Pathol..

[3] Malik T.Y., Chishti U., Aziz A.B., Sheikh I. (2016). Comparison of risk factors and survival of type 1 and type ii endometrial cancers. Pak. J. Med. Sci..

[4] Moore K.N., Fader A.N. (2011). Uterine papillary serous carcinoma. Clin. Obstet. Gynecol..

[5] Lobo F.D., Thomas E. (2016). Type ii endometrial cancers: A case series. J. Midlife Health.

[6] Horn L.C., Meinel A., Handzel R., Einenkel J. (2007). Histopathology of endometrial hyperplasia and endometrial carcinoma: An update. Ann. Diagn. Pathol..

[7] Sherman M.E., Sturgeon S., Brinton L.A., Potischman N., Kurman R.J., Berman M.L., Mortel R., Twiggs L.B., Barrett R.J., Wilbanks G.D. (1997). Risk factors and hormone levels in patients with serous and endometrioid uterine carcinomas. Mod. Pathol..

[8] Levine D.A., The Cancer Genome Atlas Research Network (2013). Integrated genomic characterization of endometrial carcinoma. Nature.

[9] Talhouk A., McConechy M.K., Leung S., Li-Chang H.H., Kwon J.S., Melnyk N., Yang W., Senz J., Boyd N., Karnezis A.N. (2015). A clinically applicable molecular-based classification for endometrial cancers. Br. J. Cancer.

[10] Talhouk A., McConechy M.K., Leung S., Yang W., Lum A., Senz J., Boyd N., Pike J., Anglesio M., Kwon J.S. (2017). Confirmation of promise: A simple, genomics-based clinical classifier for endometrial cancer. Cancer.

[11] Kommoss S., McConechy M.K., Kommoss F., Leung S., Bunz A., Magrill J., Britton H., Kommoss F., Grevenkamp F., Karnezis A. (2018). Final validation of the promise molecular classifier for endometrial carcinoma in a large population-based case series. Ann. Oncol..

[12] Raffone A., Travaglino A., Raimondo D., Boccellino M.P., Maletta M., Borghese G., Casadio P., Insabato L., Mollo A., Zullo F. (2021). Tumor-infiltrating lymphocytes and pole mutation in endometrial carcinoma. Gynecol. Oncol..

[13] Huvila J., Orte K., Vainio P., Mettala T., Joutsiniemi T., Hietanen S. (2021). Molecular subtype diagnosis of endometrial carcinoma: Comparison of the next-generation sequencing panel and proactive molecular risk classifier for endometrial cancer classifier. Hum. Pathol..

[14] Urick M.E., Bell D.W. (2019). Clinical actionability of molecular targets in endometrial cancer. Nat. Rev. Cancer.

[15] Soumerai T.E., Donoghue M.T.A., Bandlamudi C., Srinivasan P., Chang M.T., Zamarin D., Cadoo K.A., Grisham R.N., O’Cearbhaill R.E., Tew W.P. (2018). Clinical utility of prospective molecular characterization in advanced endometrial cancer. Clin. Cancer Res..

[16] Tsimberidou A.M., Iskander N.G., Hong D.S., Wheler J.J., Falchook G.S., Fu S., Piha-Paul S., Naing A., Janku F., Luthra R. (2012). Personalized medicine in a phase i clinical trials program: The md anderson cancer center initiative. Clin. Cancer Res..

[17] Tashiro H., Blazes M.S., Wu R., Cho K.R., Bose S., Wang S.I., Li J., Parsons R., Ellenson L.H. (1997). Mutations in pten are frequent in endometrial carcinoma but rare in other common gynecological malignancies. Cancer Res..

[18] Peiffer S.L., Herzog T.J., Tribune D.J., Mutch D.G., Gersell D.J., Goodfellow P.J. (1995). Allelic loss of sequences from the long arm of chromosome 10 and replication errors in endometrial cancers. Cancer Res..

[19] Risinger J.I., Hayes K., Maxwell G.L., Carney M.E., Dodge R.K., Barrett J.C., Berchuck A. (1998). Pten mutation in endometrial cancers is associated with favorable clinical and pathologic characteristics. Clin. Cancer Res..

[20] Daikoku T., Hirota Y., Tranguch S., Joshi A.R., DeMayo F.J., Lydon J.P., Ellenson L.H., Dey S.K. (2008). Conditional loss of uterine pten unfailingly and rapidly induces endometrial cancer in mice. Cancer Res..

[21] O’Hara A.J., Bell D.W. (2012). The genomics and genetics of endometrial cancer. Adv. Genom. Genet..

[22] Jani K.S., Jain S.U., Ge E.J., Diehl K.L., Lundgren S.M., Muller M.M., Lewis P.W., Muir T.W. (2019). Histone h3 tail binds a unique sensing pocket in ezh2 to activate the prc2 methyltransferase. Proc. Natl. Acad. Sci. USA.

[23] Kim K.H., Roberts C.W. (2016). Targeting ezh2 in cancer. Nat. Med..

[24] Oki S., Sone K., Oda K., Hamamoto R., Ikemura M., Maeda D., Takeuchi M., Tanikawa M., Mori-Uchino M., Nagasaka K. (2017). Oncogenic histone methyltransferase ezh2: A novel prognostic marker with therapeutic potential in endometrial cancer. Oncotarget.

[25] Bachmann I.M., Halvorsen O.J., Collett K., Stefansson I.M., Straume O., Haukaas S.A., Salvesen H.B., Otte A.P., Akslen L.A. (2006). Ezh2 expression is associated with high proliferation rate and aggressive tumor subgroups in cutaneous melanoma and cancers of the endometrium, prostate, and breast. J. Clin. Oncol..

[26] Varambally S., Dhanasekaran S.M., Zhou M., Barrette T.R., Kumar-Sinha C., Sanda M.G., Ghosh D., Pienta K.J., Sewalt R.G., Otte A.P. (2002). The polycomb group protein ezh2 is involved in progression of prostate cancer. Nature.

[27] Sashida G., Harada H., Matsui H., Oshima M., Yui M., Harada Y., Tanaka S., Mochizuki-Kashio M., Wang C., Saraya A. (2014). Ezh2 loss promotes development of myelodysplastic syndrome but attenuates its predisposition to leukaemic transformation. Nat. Commun..

[28] Ernst T., Chase A.J., Score J., Hidalgo-Curtis C.E., Bryant C., Jones A.V., Waghorn K., Zoi K., Ross F.M., Reiter A. (2010). Inactivating mutations of the histone methyltransferase gene ezh2 in myeloid disorders. Nat. Genet..

[29] Yan K.S., Lin C.Y., Liao T.W., Peng C.M., Lee S.C., Liu Y.J., Chan W.P., Chou R.H. (2017). Ezh2 in cancer progression and potential application in cancer therapy: A friend or foe?. Int. J. Mol. Sci..

[30] Fang X., Ni N., Lydon J.P., Ivanov I., Bayless K.J., Rijnkels M., Li Q. (2019). Enhancer of zeste 2 polycomb repressive complex 2 subunit is required for uterine epithelial integrity. Am. J. Pathol..

[31] Soyal S.M., Mukherjee A., Lee K.Y., Li J., Li H., DeMayo F.J., Lydon J.P. (2005). Cre-mediated recombination in cell lineages that express the progesterone receptor. Genesis.

[32] Shen X., Liu Y., Hsu Y.J., Fujiwara Y., Kim J., Mao X., Yuan G.C., Orkin S.H. (2008). Ezh1 mediates methylation on histone h3 lysine 27 and complements ezh2 in maintaining stem cell identity and executing pluripotency. Mol. Cell.

[33] Gao Y., Lin P., Lydon J.P., Li Q. (2017). Conditional abrogation of transforming growth factor-beta receptor 1 in pten-inactivated endometrium promotes endometrial cancer progression in mice. J. Pathol..

[34] Fang X., Ni N., Gao Y., Vincent D.F., Bartholin L., Li Q. (2018). A novel mouse model of testicular granulosa cell tumors. Mol. Hum. Reprod..

[35] Livak K.J., Schmittgen T.D. (2001). Analysis of relative gene expression data using real-time quantitative pcr and the 2(-delta delta c(t)) method. Methods.

[36] Wang X., Spandidos A., Wang H., Seed B. (2012). Primerbank: A pcr primer database for quantitative gene expression analysis, 2012 update. Nucleic Acids Res..

[37] Naganuma T., Takagi S., Kanetake T., Kitamura T., Hattori S., Miyakawa T., Sassa T., Kihara A. (2016). Disruption of the sjogren-larsson syndrome gene aldh3a2 in mice increases keratinocyte growth and retards skin barrier recovery. J. Biol. Chem..

[38] Blanco-Aparicio C., Renner O., Leal J.F.M., Carnero A. (2007). Pten, more than the akt pathway. Carcinogenesis.

[39] Blaisdell A., Crequer A., Columbus D., Daikoku T., Mittal K., Dey S.K., Erlebacher A. (2015). Neutrophils oppose uterine epithelial carcinogenesis via debridement of hypoxic tumor cells. Cancer Cell.

[40] Cheng Y.H., Utsunomiya H., Pavone M.E., Yin P., Bulun S.E. (2011). Retinoic acid inhibits endometrial cancer cell growth via multiple genomic mechanisms. J. Mol. Endocrinol..

[41] Wu L., Saxena S., Awaji M., Singh R.K. (2019). Tumor-associated neutrophils in cancer: Going pro. Cancers.

[42] Korkmaz B., Horwitz M.S., Jenne D.E., Gauthier F. (2010). Neutrophil elastase, proteinase 3, and cathepsin g as therapeutic targets in human diseases. Pharmacol. Rev..

[43] Pijnenborg J.M.A., Wijnakker M., Hagelstein J., Delvoux B., Groothuis P.G. (2007). Hypoxia contributes to development of recurrent endometrial carcinoma. Int. J. Gynecol. Cancer.

[44] Mahiddine K., Blaisdell A., Ma S., Crequer-Grandhomme A., Lowell C.A., Erlebacher A. (2020). Relief of tumor hypoxia unleashes the tumoricidal potential of neutrophils. J. Clin. Investig..

[45] Tangen I.L., Werner H.M., Berg A., Halle M.K., Kusonmano K., Trovik J., Hoivik E.A., Mills G.B., Krakstad C., Salvesen H.B. (2014). Loss of progesterone receptor links to high proliferation and increases from primary to metastatic endometrial cancer lesions. Eur. J. Cancer.

[46] Kim J.J., Chapman-Davis E. (2010). Role of progesterone in endometrial cancer. Semin. Reprod. Med..

[47] Chung H.H., Or Y.Z., Shrestha S., Loh J.T., Lim C.L., Ong Z., Woo A.R.E., Su I.H., Lin V.C.L. (2017). Estrogen reprograms the activity of neutrophils to foster protumoral microenvironment during mammary involution. Sci. Rep..

[48] Vazquez Rodriguez G., Abrahamsson A., Jensen L.D., Dabrosin C. (2017). Estradiol promotes breast cancer cell migration via recruitment and activation of neutrophils. Cancer Immunol. Res..

[49] Maxwell G.L., Risinger J.I., Gumbs C., Shaw H., Bentley R.C., Barrett J.C., Berchuck A., Futreal P.A. (1998). Mutation of the pten tumor suppressor gene in endometrial hyperplasias. Cancer Res..

[50] Gu Y., Zhang J., Guan H. (2017). Expression of ezh2 in endometrial carcinoma and its effects on proliferation and invasion of endometrial carcinoma cells. Oncol. Lett..

[51] Roh J.W., Choi J.E., Han H.D., Hu W., Matsuo K., Nishimura M., Lee J.S., Kwon S.Y., Cho C.H., Kim J. (2020). Clinical and biological significance of ezh2 expression in endometrial cancer. Cancer Biol. Ther..

[52] Ihira K., Dong P., Xiong Y., Watari H., Konno Y., Hanley S.J., Noguchi M., Hirata N., Suizu F., Yamada T. (2017). Ezh2 inhibition suppresses endometrial cancer progression via mir-361/twist axis. Oncotarget.

[53] Kikuchi J., Koyama D., Wada T., Izumi T., Hofgaard P.O., Bogen B., Furukawa Y. (2015). Phosphorylation-mediated ezh2 inactivation promotes drug resistance in multiple myeloma. J. Clin. Investig..

[54] Mallen-St Clair J., Soydaner-Azeloglu R., Lee K.E., Taylor L., Livanos A., Pylayeva-Gupta Y., Miller G., Margueron R., Reinberg D., Bar-Sagi D. (2012). Ezh2 couples pancreatic regeneration to neoplastic progression. Genes Dev..

[55] Nanjappa M.K., Mesa A.M., Medrano T.I., Jefferson W.N., DeMayo F.J., Williams C.J., Lydon J.P., Levin E.R., Cooke P.S. (2019). The histone methyltransferase ezh2 is required for normal uterine development and function in mice. Biol. Reprod..

[56] Mesa A.M., Mao J.D., Nanjappa M.K., Medrano T.I., Tevosian S., Yu F.H., Kinkade J., Lyu Z., Liu Y., Joshi T. (2020). Mice lacking uterine enhancer of zeste homolog 2 have transcriptomic changes associated with uterine epithelial proliferation. Physiol. Genom..

[57] Mesa A.M., Mao J., Medrano T.I., Bivens N.J., Jurkevich A., Tuteja G., Cooke P.S., Rosenfeld C.S. (2021). Spatial transcriptomics analysis of uterine gene expression in enhancer of zeste homolog 2 conditional knockout micedagger. Biol. Reprod..

[58] Xie Y., Naizabekov S., Chen Z., Tokay T. (2016). Power of pten/akt: Molecular switch between tumor suppressors and oncogenes. Oncol. Lett..

[59] Sirohi V.K., Medrano T.I., Mesa A.M., Kannan A., Bagchi I.C., Cooke P.S. (2022). Regulation of akt signaling in mouse uterus. Endocrinology.

[60] Kruger P., Saffarzadeh M., Weber A.N., Rieber N., Radsak M., von Bernuth H., Benarafa C., Roos D., Skokowa J., Hartl D. (2015). Neutrophils: Between host defence, immune modulation, and tissue injury. PLoS Pathog..

[61] Masucci M.T., Minopoli M., Carriero M.V. (2019). Tumor associated neutrophils. Their role in tumorigenesis, metastasis, prognosis and therapy. Front. Oncol..

[62] Wang Y., Hou N., Cheng X., Zhang J., Tan X., Zhang C., Tang Y., Teng Y., Yang X. (2017). Ezh2 acts as a tumor suppressor in kras-driven lung adenocarcinoma. Int. J. Biol. Sci..

[63] Gan L., Yang Y., Li Q., Feng Y., Liu T., Guo W. (2018). Epigenetic regulation of cancer progression by ezh2: From biological insights to therapeutic potential. Biomark Res..

[64] Shen M., Hu P., Donskov F., Wang G., Liu Q., Du J. (2014). Tumor-associated neutrophils as a new prognostic factor in cancer: A systematic review and meta-analysis. PLoS ONE.

[65] Cunha G.R., Kurita T., Cao M., Shen J., Robboy S., Baskin L. (2017). Molecular mechanisms of development of the human fetal female reproductive tract. Differentiation.

[66] McCaffrey L.M., Macara I.G. (2011). Epithelial organization, cell polarity and tumorigenesis. Trends Cell Biol..

[67] Filant J., DeMayo F.J., Pru J.K., Lydon J.P., Spencer T.E. (2014). Fibroblast growth factor receptor two (fgfr2) regulates uterine epithelial integrity and fertility in mice. Biol. Reprod..

[68] Rodriguez A.C., Blanchard Z., Maurer K.A., Gertz J. (2019). Estrogen signaling in endometrial cancer: A key oncogenic pathway with several open questions. Horm. Cancer.

[69] Lindberg M.E., Stodden G.R., King M.L., MacLean J.A., Mann J.L., DeMayo F.J., Lydon J.P., Hayashi K. (2013). Loss of cdh1 and pten accelerates cellular invasiveness and angiogenesis in the mouse uterus. Biol. Reprod..

[70] Fedorko A.M., Kim T.H., Broaddus R., Schmandt R., Chandramouli G.V.R., Kim H.I., Jeong J.W., Risinger J.I. (2020). An immune competent orthotopic model of endometrial cancer with metastasis. Heliyon.

[71] Wang X., Wendel J.R.H., Emerson R.E., Broaddus R.R., Creighton C.J., Rusch D.B., Buechlein A., DeMayo F.J., Lydon J.P., Hawkins S.M. (2020). Pten and dicer1 loss in the mouse uterus causes poorly differentiated endometrial adenocarcinoma. Oncogene.

[72] Koppens M.A., Tanger E., Nacerddine K., Westerman B., Song J.Y., van Lohuizen M. (2017). A new transgenic mouse model for conditional overexpression of the polycomb group protein ezh2. Transgenic Res..

[73] Ideker T., Galitski T., Hood L. (2001). A new approach to decoding life: Systems biology. Annu. Rev. Genom. Hum. Genet..

[74] Qiu J., Chen K., Zhong C., Zhu S., Ma X. (2021). Network-based protein-protein interaction prediction method maps perturbations of cancer interactome. PLoS Genet..

[75] Kim M., Park J., Bouhaddou M., Kim K., Rojc A., Modak M., Soucheray M., McGregor M.J., O’Leary P., Wolf D. (2021). A protein interaction landscape of breast cancer. Science.

[76] Pilot-Storck F., Chopin E., Rual J.F., Baudot A., Dobrokhotov P., Robinson-Rechavi M., Brun C., Cusick M.E., Hill D.E., Schaeffer L. (2010). Interactome mapping of the phosphatidylinositol 3-kinase-mammalian target of rapamycin pathway identifies deformed epidermal autoregulatory factor-1 as a new glycogen synthase kinase-3 interactor. Mol. Cell Proteom..

[77] Pane K., Affinito O., Zanfardino M., Castaldo R., Incoronato M., Salvatore M., Franzese M. (2020). An integrative computational approach based on expression similarity signatures to identify protein-protein interaction networks in female-specific cancers. Front. Genet..

[78] Yap T.A., Winter J.N., Giulino-Roth L., Longley J., Lopez J., Michot J.M., Leonard J.P., Ribrag V., McCabe M.T., Creasy C.L. (2019). Phase i study of the novel enhancer of zeste homolog 2 (ezh2) inhibitor gsk2816126 in patients with advanced hematologic and solid tumors. Clin. Cancer Res..

[79] Italiano A., Soria J.C., Toulmonde M., Michot J.M., Lucchesi C., Varga A., Coindre J.M., Blakemore S.J., Clawson A., Suttle B. (2018). Tazemetostat, an ezh2 inhibitor, in relapsed or refractory b-cell non-hodgkin lymphoma and advanced solid tumours: A first-in-human, open-label, phase 1 study. Lancet Oncol..

