# Supplementary material for: EZH2 and Endometrial Cancer Development: Insights from a Mouse Model
Source: Cells. 2022 Mar 7;11(5):909. doi: 10.3390/cells11050909 (PMC8909840; doi:10.3390/cells11050909)
Supplement: Supplementary file 1 [file cells-11-00909-s001.zip › cells-1590131-supplementary.pdf]

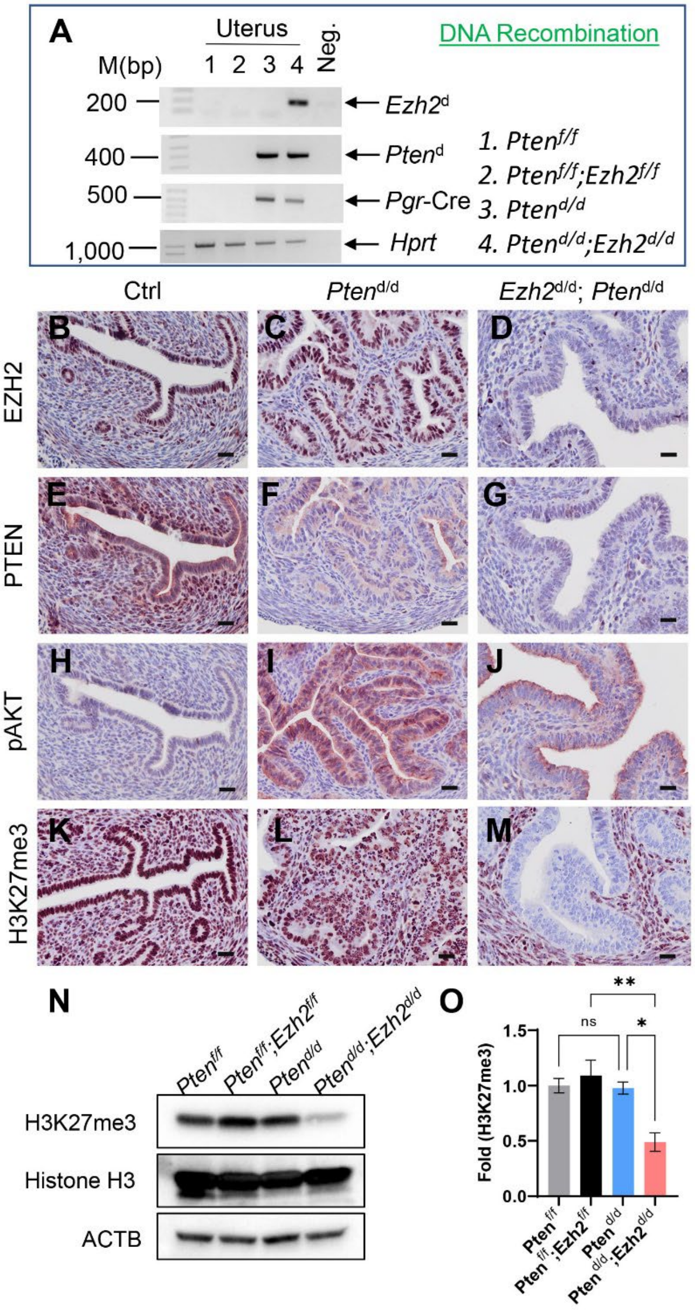

Figure S1. Generation and validation of mice with conditional deletion of *Ezh2* and *Pten*. (**A**) Analysis of DNA recombination of *Ezh2* and *Pten* alleles in the uteri of *Pten*$^{f/f}$ (Ctrl), *Pten*$^{d/d}$, and *Pten*$^{d/d}$; *Ezh2*$^{d/d}$ mice at 2 weeks of age. n = 3. (**B**-**M**) Immunostaining of EZH2 (B-D), PTEN (E-G), pAKT (H-J), and H3K27me3 (K-M) using uteri from 2-week-old *Pten*$^{f/f}$ (Ctrl), *Pten*$^{d/d}$, and *Pten*$^{d/d}$; *Ezh2*$^{d/d}$ mice. At least three independent samples were examined for each genotype. Scale bar = 20 μm (B-M). (**N**) Western blot analysis of H3K27me3 in the uteri from 2-week-old *Pten*$^{f/f}$, *Pten*$^{f/f}$; *Ezh2*$^{f/f}$, *Pten*$^{d/d}$, and *Pten*$^{d/d}$; *Ezh2*$^{d/d}$ mice. Histone H3 was used as an internal control. (**O**) Quantification of western blot. Data were normalized to the control group (100%). n = 4. *P < 0.05 and **P < 0.01. ns, not significant.

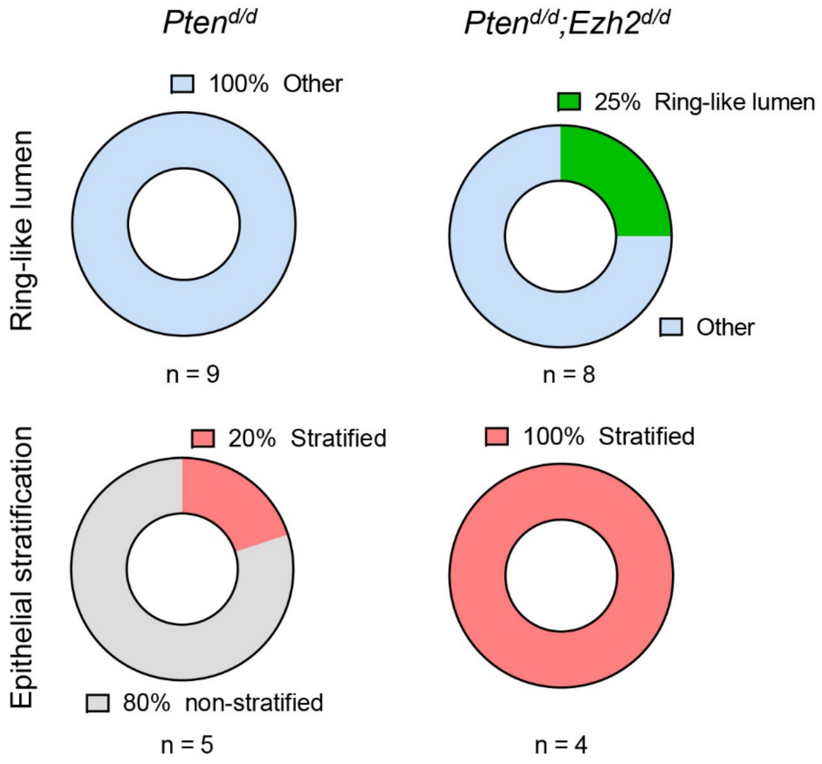

Figure S2. A summary of uterine histological features in *Pten*$^{d/d}$ and *Pten*$^{d/d}$; *Ezh2*$^{d/d}$ mice at 1 month of age. The percentage of mice is used for the pie chart.

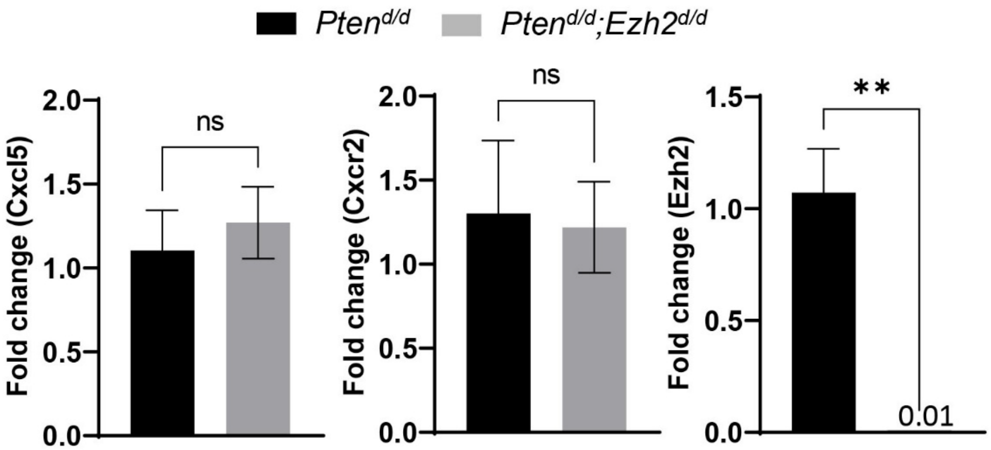

Figure S3. Levels of *Cxcl5*, *Cxcr2*, and *Ezh2* transcripts in uterine epithelial cells isolated from *Pten*$^{d/d}$ and *Pten*$^{d/d}$; *Ezh2*$^{d/d}$ mice. Data are mean ± s.e.m. n = 4-5. $^{**}P < 0.01$. ns, not significant.

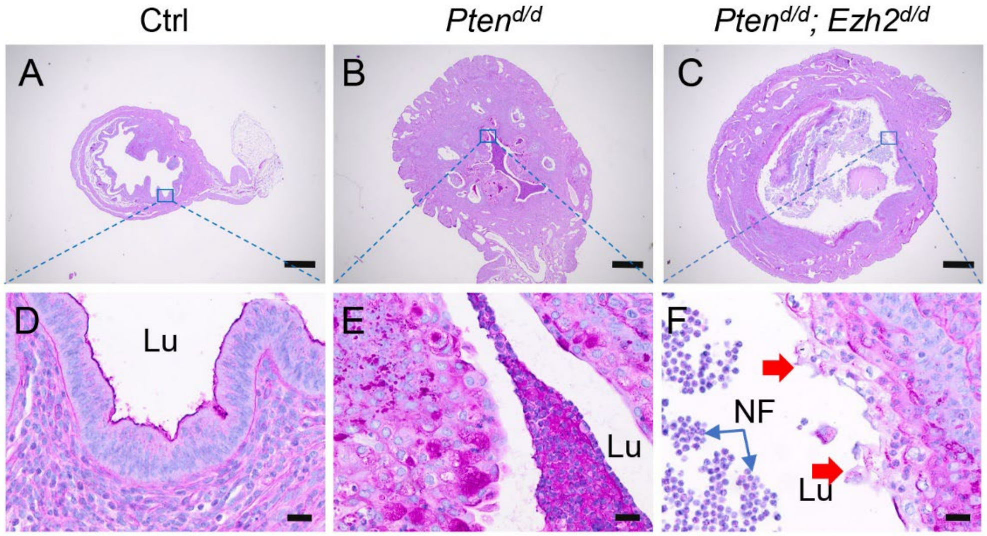

Figure S4. Intraluminal neutrophil infiltration in the uteri of *Pten*$^{d/d}$ and *Pten*$^{d/d}$*; Ezh2*$^{d/d}$ mice. (**A**-**F**) PAS staining of uteri from 9-week-old Ctrl, *Pten*$^{d/d}$, and *Pten*$^{d/d}$*; Ezh2*$^{d/d}$ mice. Panels (D-F) are high power images of the boxed areas of panels (A-C), respectively. At least three independent samples were examined for each genotype. Red arrows indicate the sloughing epithelia. Lu, lumen. NF, neutrophils. Scale bar = 20 μm (D-F) and 500 μm (A-C).

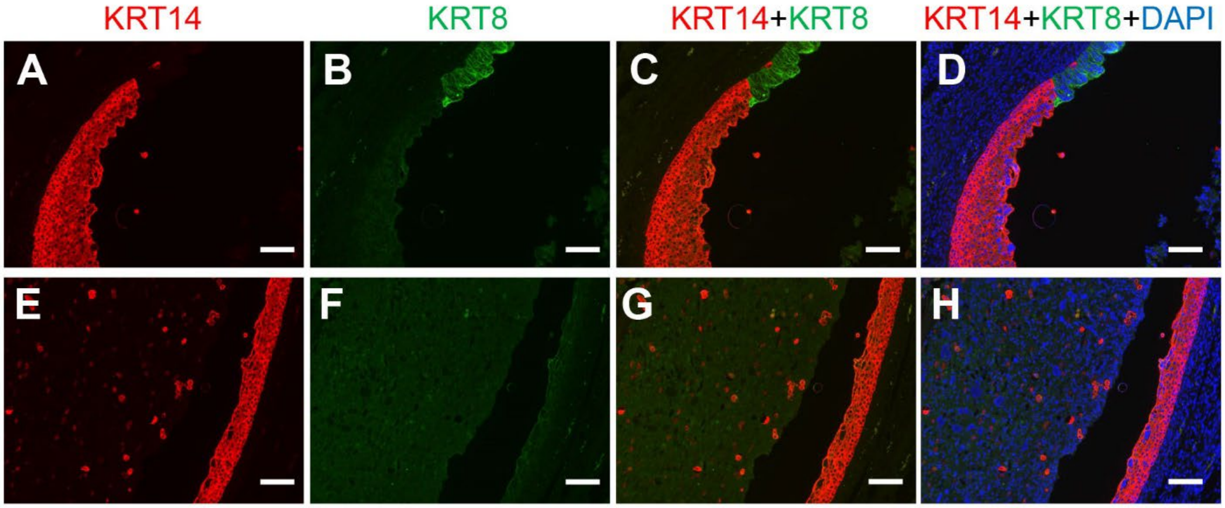

Figure S5. Immunofluorescence of KRT14 and KRT8 using uteri from 1-month-old *Pten*$^{d/d}$; *Ezh2*$^{d/d}$ mice. DAPI was used to counterstain the nuclei. Scale bar = 100 μm.

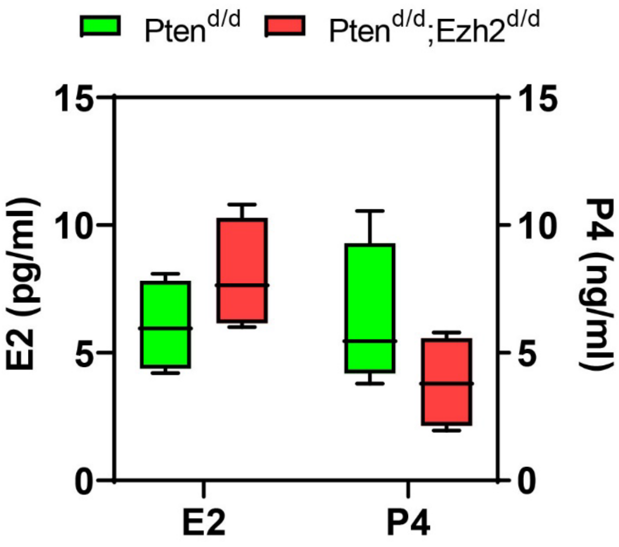

Figure S6. Serum estrogen and progesterone levels in *Pten*$^{d/d}$ and *Pten*$^{d/d}$*; Ezh2*$^{d/d}$ mice. Sera were collected from mice at the age of 9 weeks. n = 4.
